# Vaccine uptake and hesitancy among Black people: A scoping review

**DOI:** 10.14745/ccdr.v52i03a02

**Published:** 2026-03-31

**Authors:** Folajinmi Oluwasina, Salwa Musa, Mary Olukotun, Folakemi Ojo, Omolara Sanni, Lynda Djoutsa, Modupe Tunde-Byass, Andre Renzaho, Upton Allen, Bukola Salami

**Affiliations:** 1 Faculty of Nursing, University of Alberta, Edmonton, AB; 2 Department of Science, Red Deer Polytechnic, Red Deer, AB; 3 Obstetrics and Gynaecology, North York General Hospital, North York, ON; 4 School of Medicine, Western Sydney University, Penrith, Australia; 5 Department of Medicine, University of Toronto, Toronto, ON; 6 Department of Paediatrics, The Hospital for Sick Children, Toronto, ON; 7 Cumming School of Medicine, University of Calgary, Calgary, AB

**Keywords:** vaccine, uptake, hesitancy, Black people

## Abstract

**Background:**

Vaccination is one of the most cost-effective ways to prevent disease, yet vaccine hesitancy remains a threat to the progress made in tackling vaccine preventable diseases. Black communities have a history of being subjects of unethical research, victims of implicit bias, mistreated by healthcare professionals, and denied access to medical assistance. This study aims to examine vaccine uptake and hesitancy among Black people.

**Methods:**

A scoping review was conducted in 11 bibliographic databases to identify relevant peer-reviewed studies. Articles were screened by two reviewers, with a third resolving conflicts where necessary. Data were extracted from eligible studies and findings were narratively summarized. A PRISMA checklist was adopted, followed by data extraction with the findings then collated, summarized, and reported.

**Results:**

A total of 101 articles (77 quantitative, 16 qualitative, 3 randomized clinical trials, and 5 mixed methods studies) were included in the final analysis. Among these, 95.1% and 4.9% reported findings from North America and Europe, respectively. This review revealed that misinformation affects the acceptability of vaccination programs. Vaccine hesitancy among Black communities is often rooted in fears of potential side effects and long-term consequences. Parental consent was noted as a crucial issue, and the belief that children should not be offered vaccinations without parental consent was indicated as a factor affecting vaccine uptake.

**Conclusion:**

Vaccine hesitancy continues to have a significant impact on global health. Government policies that promote vaccine uptake would help to reduce vaccine hesitancy and maintain high coverage among Black people.

## Introduction

Vaccination is one of the most cost-effective ways of avoiding disease ( ((1))), yet vaccine hesitancy is a threat to the progress made in tackling vaccine preventable diseases. Vaccine hesitancy, as defined by the World Health Organization (WHO), is a delay in acceptance or refusal of vaccination despite the availability of vaccination services ( ((2))). In 2019, the WHO classified vaccine hesitancy as one of the top 10 threats to global health and stated that the degree of vaccine hesitancy can vary depending on a variety of factors, including the type of vaccine and target population ( ((2))). For example, in the United States (US), the Advisory Committee on Immunization Practices has recommended influenza vaccines for all people above six months of age, but the rate of influenza vaccination coverage for adults and children is below the Healthy People 2020 initiative’s target of 70% ( ((3))).

Vaccine hesitancy is rapidly increasing and often influenced by factors such as geographical accessibility of vaccines, affordability, cultural influence of the population, confidence or trust in the vaccine, safety of the vaccine, and the system that delivers them ( ((4))). For example, the experience of discrimination in health care may contribute to medical distrust, which is associated with a lower likelihood of receiving preventive health service ( ((5))). Black communities have a history of being mistreated by healthcare professionals and being denied access to medical assistance ( ((4))). In a study conducted in 2019, Jamila *et al.* found the American healthcare system is beset with inequalities that have a disproportionate impact on people of colour and other marginalized groups. These inequalities contribute to gaps in health insurance coverage, uneven access to services, and poorer health outcomes among certain populations. Black adults are significantly less willing to get vaccinated than adults who are White or of other races ( ((6))). Decision-making around vaccination entails a complex mix of cultural, psychosocial, spiritual, political, and cognitive factors ( ((7))). Reasons for vaccine hesitancy generally fit into three categories: lack of confidence (in effectiveness, safety, the system, or policy makers), complacency, and lack of convenience (with respect to the availability, accessibility, and appeal of immunization services, including time, place, language, and cultural contexts) ( ((8))).

Socioeconomic status can affect vaccine uptake. In the US, Black people are more likely to live in poor or low-income neighbourhoods and are less likely to have health insurance than people of other races ( ((9))). For example, low-income levels and lack of medical insurance are associated with lower rates of human papillomavirus (HPV) vaccine initiation in young Black women ( ((10))). Reduced access to vaccination centres in Black communities compared to White communities is also associated with lower uptake of vaccines ( ((11))).

This scoping review seeks to synthesize what is known in the literature on factors that contribute to vaccine hesitancy and uptake among Black people.

## Methods

This scoping review was planned and conducted in adherence with the Preferred Reporting Items for Systematic Reviews and Meta-Analyses Extension for Scoping Reviews (PRISMA-ScR) statement with a focus on health equity ( ((12))). The study adopted a comprehensive search strategy that allowed reproducibility, reliability, and transparency on the current state of literature. The review was conducted in five stages, as described below.

### Stage 1: Developing the research question

The research question was: “What is known about vaccine uptake and hesitancy among Black people?”

### Stage 2: Identifying the relevant studies

**Figure 1:**
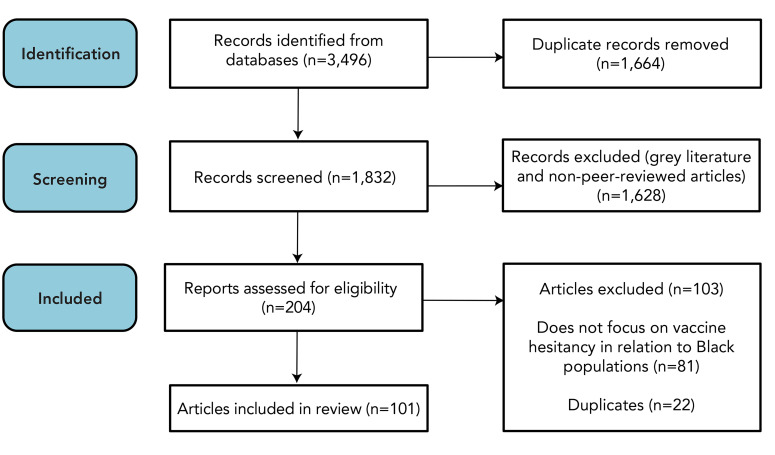
PRISMA study selection diagram Abbreviation: PRISMA, Preferred Reporting Items for Systematic Reviews and Meta-Analyses

Original peer-reviewed articles from database inception until July 2023 were obtained from systematic searches of several electronic bibliographic databases: MEDLINE (1946–present), Embase (1974–present), PsycInfo (1806–present), Global Health (1910–present), and HealthSTAR (1966–present) via OVID; Cumulative Index to Nursing and Allied Health Literature (CINAHL) (1936–present) and Environment Complete (1950–present) via EBSCOhost; Scopus (1976–present) via Elsevier; Sociological Abstracts (1952–present) and Dissertations and Theses Global (1861–present) via ProQuest; and Cochrane Library (1993–present) via Wiley. These databases were examined using a mixture of natural language vocabulary and controlled terms (subject headings) wherever available, with both derived from three main concepts: 1) vaccine uptake, 2) vaccine hesitancy, and 3) Black population. To increase search sensitivity, publication date and study type restrictions were not applied. In total, 3,496 records were identified through database searching. Duplicate records (n=1,664) were automatically removed upon importation into the systematic review management software, Covidence. The full search strategy has been attached as **Figure 1**.

### Stage 3: Article selection

Based on eligibility criteria, six research assistants with backgrounds in health sciences screened the articles for selection. The first selection was the screening of the title and abstract and the second screening was a full-text review. All conflicts generated through the screening stages between the reviewers were resolved by a third reviewer. Each eligible article met the following inclusion criteria: 1) focus on vaccine uptake and/or 2) focus on vaccine hesitancy, and 3) focus on the Black population.

Only primary research published in peer-reviewed journals was included. Grey literature and non-peer-reviewed articles were excluded. No language or publication restrictions were applied. The first screening removed an additional 1,628 duplicates, leaving 204 articles for full-text review. Full-text review resulted in the exclusion of 81 articles that did not meet the inclusion criteria and 22 additional duplicate articles, resulting in a total of 101 articles eligible for inclusion in this scoping review. All were written in English. We also reviewed the reference lists of included articles but found no additional studies that met our inclusion criteria. This information is summarized in detail in the PRISMA flow diagram (Figure 1).

### Stage 4: Data charting and data extraction

The following information was extracted from each of the 101 articles: author(s) name, year of publication, country of study, study design, sample size, age, type of vaccine, findings, and conclusion.

### Stage 5: Collating, summarizing, and reporting the results

The characteristics and results reported in each included article are summarily described. An overview of existing evidence relating to vaccine uptake and/or hesitancy among Black people is presented.

## Results

### Characteristics of included studies

**Figure 2:**
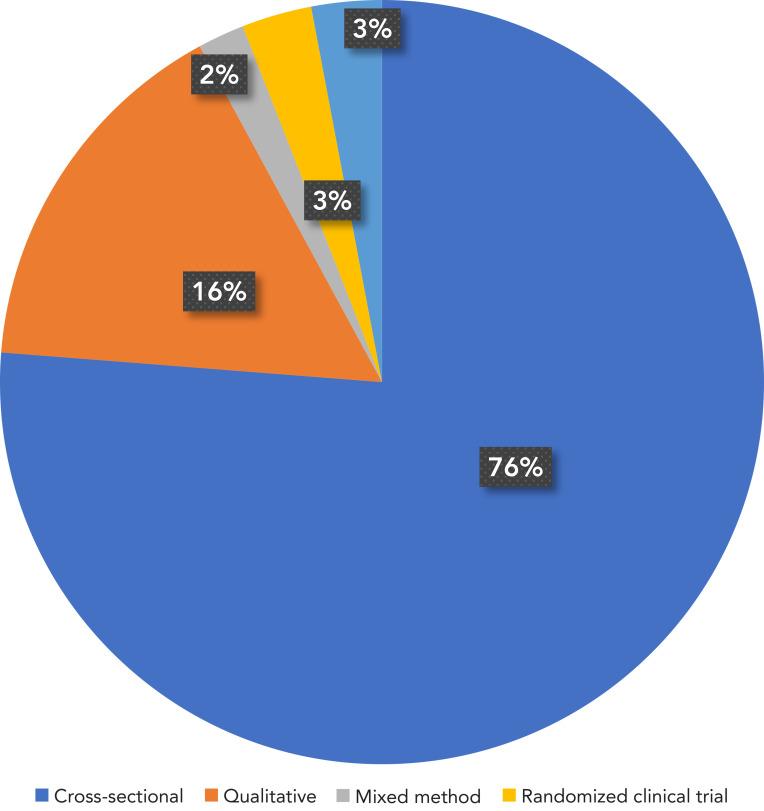
Study methods used in the included articles

The characteristics of the 101 articles included in this review are presented in the **Appendix**, Table S1. The majority of the articles, 76% (n=77), were cross-sectional studies (retrospective and descriptive), while 16% (n=16) were qualitative studies, 2% (n=2) were mixed methods, 3% (n=3) were randomized clinical trials, and 3% (n=3) did not specify the research methodology (**Figure 2**).

The study population size ranged from 20 participants to 2.2 million participants. Study participant ages, where reported, ranged from 18 months to >75 years. The articles assessed different types of vaccines, including COVID-19, HPV, pneumococcal, influenza, and measles, mumps, rubella (MMR).

Sentiments toward vaccination vary within the Black community. Overall, all studies identified lower rates of vaccine uptake among Black populations regardless of geographic location. Distinctive themes emerged from this review that highlighted the structural, cognitive dissonance, and behavioural barriers that facilitate vaccine hesitancy within Black populations. Barriers to vaccine uptake were related to: 1) interactions with healthcare providers, 2) cost and insurance status, 3) convenience of immunization, and 4) general knowledge related to vaccine preventable diseases. Furthermore, macro-level themes related to vaccine hesitancy included: 1) distrust in the vaccine development process, 2) safety and efficacy, 3) perceived risk, 4) historical considerations, 5) distrust in the government, and 6) cultural, religious, and family considerations.

### Barriers to vaccine uptake in Black communities

#### Interactions with healthcare providers

Thirteen studies reported on interactions with healthcare providers and related impacts on vaccine uptake. Interactions with healthcare providers have been shown to affect vaccine uptake within Black populations. Healthcare providers who discussed vaccination and offered vaccination as a routine practice contributed to higher vaccine uptake ( ((11,13–18))). Decreased vaccine uptake occurred when healthcare providers did not initiate discussions during appointments because patients were reluctant to initiate such conversations ( ((19–21))). In addition, male healthcare providers were less likely than female healthcare providers to discuss HPV vaccination with Black patients ( ((22))). Healthcare providers were viewed as essential to supporting vaccine uptake and were considered the most influential external factor in vaccination decisions. Indeed, the healthcare provider is the most influential source of information and recommendations promoting vaccine uptake within a client’s plan of care and decision-making process ( ((23))). Trust is a value measure within the relationship between the healthcare provider and their patients, and healthcare providers were viewed as the most trusted sources of information relating to vaccines ( ((24))). A therapeutic relationship between the patient and healthcare provider that fosters discussions related to vaccinations was seen as essential for promoting vaccination and decreasing the occurrence of vaccine preventable diseases within Black communities.

#### Cost and insurance status

Accessibility, affordability, and willingness to pay are factors that influence vaccine uptake. Eleven studies reported on the impact of vaccination cost and number of doses on vaccine uptake. Insurance status and fee for services directly affected vaccination, completion of vaccination series, and overall vaccine uptake ( ((25–31))). Furthermore, socioeconomic status and income were also important; perceived lack of ability to pay can influence the vaccination decision-making process and vaccination uptake ( ((32,33))). Nan *et al.* (2016) reported an increase (32.7%) in vaccine acceptance occurred within the Black community when vaccinations were offered free of charge.

#### Convenience of immunization

Numerous factors contribute to the convenience of vaccination, including the availability of vaccines and accessibility of vaccination clinics. In two studies ( ((34,35))), line-ups, waiting times, and multiple appointments were cited as inconveniences for individuals who did not receive vaccinations. Factors that facilitated accessibility were cited as positive contributors to vaccine uptake, and included considerations such as minimizing multiple appointments, decreased time spent, and short lineups.

#### General knowledge related to vaccine preventable diseases

Twelve studies investigated the link between knowledge about vaccination and vaccine uptake. Lack of knowledge related to vaccination and vaccine preventable diseases impacts the client’s perceived risk and need for vaccination. The success of current immunization programs has created a paradoxical effect resulting in decreased public awareness of the negative outcomes related to vaccine preventable diseases. Limited knowledge related to vaccine preventable diseases and the availability of vaccines impacts vaccine acceptance ( ((30,35–42))). Misinformation also affects the acceptability of vaccination programs. As shown by Cooper *et al.* (2017), HPV and HPV vaccination awareness among Black men was low, contributing to low vaccine uptake. Of the men surveyed, 50% had not heard of HPV and 53% were unaware of the vaccine. In conjunction with the lack of knowledge of vaccine preventable diseases was the assumption that HPV vaccination was required for sexual activity. This behavioural association discouraged vaccination of children and adolescents as a preventive measure due to the limited discussions within parental dyads to initiate the vaccination series ( ((26))). When knowledge was shared with clients, a positive association existed between willingness to vaccinate a daughter or son based on the newly acquired understanding ( ((43–45))). Participant health literacy levels were a critical factor that influenced understanding. Lower levels of health literacy had a negative impact on vaccine uptake, as clients were skeptical and fearful of the vaccination programs being offered ( ((46))). The perceived low risk and limited knowledge related to vaccine preventable diseases were evident within geographically diverse Black communities ( ((47,48))). Overall knowledge and health literacy are important aspects of vaccine acceptability that impact the perceived value of vaccines and necessity of vaccination within Black communities.

### Themes related to vaccine hesitancy in Black communities

#### Distrust in the vaccine development process

Twelve studies reported on vaccine hesitancy related to the vaccine development process. Black participants reported being hesitant to new vaccinations offered. This hesitancy was often tied to limited knowledge of the historical impact of vaccine preventable diseases and the history of vaccine development ( ((21,24))). Reluctance toward new vaccines led individuals to prefer waiting for further research greater public awareness before accepting them ( ((21,24))). Fears were often rooted in potential side effects and long-term impacts ( ((41,49–51))). Furthermore, within Black communities, mistrust toward expedited vaccination development and trials with emerging vaccines contributed to fears that new vaccines were rushed and therefore unsafe ( ((41,49,52–56))). Such feelings were also linked to vaccine testing and willingness to get vaccinated, in particular, thoughts that testing of the COVID-19 vaccine was rushed compared to historical time frames ( ((2,57))).

#### Safety and efficacy

Fifteen studies investigated linkages between vaccine safety and efficacy and vaccination uptake. Vaccine acceptability is often attributed to aspects of vaccine safety, necessity, and effectiveness ( ((58))). Black participants expressed concerns regarding the short- and long-term effects of the vaccines ( ((2,5,19,27,35,40,42,43,55,59–61))). Participants expressed concerns about vaccinating their daughters against HPV, citing they were too young and that they were worried about the one-size-fits-all vaccination approach ( ((25))). A risk-benefit trade-off between vaccines and vaccine preventable diseases is impacted by the perception of vaccine safety. Stern *et al.* (2021) highlighted that Black participants viewed the COVID-19 vaccine as unsafe and a greater safety risk than acquiring COVID-19. Weaver *et al.* (2013) found efficacy had the greatest impact on acceptability to HIV vaccine trials with Black participants, with the acceptability of a high (99%) efficacy vaccine being significantly greater than for a 50% efficacy vaccine ( ((24))). Vaccine efficacy is supported by education from trusted sources, such as healthcare providers and government bodies, while reduced trust in health information from government bodies is associated with a decrease in perceived vaccine efficacy ( ((62))). Complacency was found to be related to perceived low disease risk and, when coupled with concerns related to vaccine efficacy, resulted in decreased vaccine uptake ( ((52))).

#### Perceived risk

Eleven studies investigated the perceived risk of negative outcomes related to vaccination. The perceived risk of acquiring vaccine preventable diseases has been shown to be directly linked to vaccine uptake. One study ( ((63))) found that the most common reason for not being vaccinated was a low perception of personal risk for vaccine preventable disease, the belief that healthy behaviours were sufficient to mitigate the risk. Furthermore, decreased vaccination was due to the perception was that they would not become seriously ill if they acquired the disease ( ((64–66))). Perceived risk was linked to limited knowledge related to vaccine preventable disease and complications ( ((25,27,52,61,65,67))).

#### Historical considerations

Two studies explored historical practices in health research within the Black community and their impact on vaccine hesitancy. Participants expressed considerable and well-founded mistrust of the medical establishment, scientific research communities, and pharmaceutical companies, based on their knowledge of historic mistreatment and lack of representation in studies that adversely affected Black patients and participants ((68)). Participants reported that their overall mistrust of these entities made them less willing to consider getting a COVID-19 vaccine, no matter how safe it was proven to be ( ((2,69))).

#### Distrust in the government

Seven studies investigated the relationship between trust in government and vaccination uptake. Trust in government agencies regarding vaccination development and programs has been shown to impact vaccine acceptance. Black participants indicated that their vaccine hesitancy was directly related to distrust in the government ( ((2,9,52,58,70,71))). Political involvement within the vaccination development process (i.e., vaccine approval) also created mistrust within Black communities. Moreover, the environment associated with political and associated racial injustice in the US, further decreased confidence in vaccination related to COVID-19 ( ((72))). Mupandawana *et al.* (2016) found that participants had a general distrust of Western societies and vaccines associated with them. The lack of trust in government agencies increased the belief in conspiracy theories, resulting in the use of naturalism as an alternative to vaccination ( ((40))).

#### Cultural, religion, and family considerations

Five studies investigated the cultural and religious implications of vaccine uptake. Culture has an impact on the decision-making process related to vaccination. Pierre Joseph *et al.* (2014) found Black family structures featured limited parent-adolescent communication about sexual activity and, for African American and Haitian men in particular, this resulted in decreased vaccination. Furthermore, Galbraith-Gyan *et al.* (2019) found the health behaviour and decision-making of African participants had deep cultural roots ( ((52))). Parental consent was seen as vital for vaccine acceptance, and the belief that children should not be offered vaccinations without parental consent was indicated to impact vaccine uptake ( ((35,69))). Furthermore, the vaccination decision-making process in Black communities may include members outside of the traditional nuclear family, such as cultural leaders, elders, and grandparents ( ((10))).

Religious values and cultural norms influenced vaccine decision-making in Black families, with fathers acting as the ultimate decision-makers ( ((52))). The risk of judgment from cultural and religious groups with which people identify impacted enrolment within vaccine trials due to potential stigmatization related to disease status ( ((40))). Furthermore, vaccination status, particularly for HPV, was seen to be linked with sexual activity, leading some parents of adolescents to express fear that vaccination might encourage promiscuity ( ((52,73))).

## Discussion

This scoping review indicates vaccine uptake is influenced by multi-level factors, including the influence of the healthcare provider; convenience related to cost, insurance status, or the number of doses and visits required; and knowledge or lack of knowledge pertaining to vaccinations. Furthermore, the findings show vaccine hesitancy is influenced by factors such as the perception that the vaccine development process was rushed or too novel; concerns around safety and efficacy; perceived risk; hesitancy based on unethical historical practices in research toward the Black community; mistrust in government; and cultural, religious, or family structure. Collectively, these identified themes highlight vaccine uptake and hesitancy in Black communities as dynamic concerns with significant implications with respect to both short- and long-term health outcomes of Black people. Considering the ongoing threat of COVID-19, the high rates of infection and mortality in Black communities, as well as the increased prevalence of COVID-19 vaccine hesitancy among racial and ethnic minority groups ( ((74))), this paper is timely and relevant in its contribution to current public health discourse.

The factors identified as barriers to vaccine uptake are reflective of health service barriers experienced by Black communities, particularly those related to accessing primary care and other preventive health services ( ((75–78))). In a qualitative study on access to care as a barrier to Black women’s use of mammography, location and transportation, lack of insurance, healthcare costs, inadequate information, wait times for an appointment, and failure of the healthcare provider to disclose mammography information or recommend mammography were some of the notable barriers shared by the participants ( ((75))). Similar barriers are documented for colorectal cancer screening ( ((77))), primary care use among African American men ( ((78))), and HPV immunization for Black adolescents ( ((76))).

For Black populations, the prevalence of conversations with healthcare providers about vaccines is disproportionate to levels of vaccine awareness; even in circumstances in which Black people report higher rates of vaccine-related conversations with their healthcare providers than their White counterparts, they conversely report lower rates of vaccine awareness ( ((77,79))), raising concerns about the quality of conversations between patients and providers. Unfortunately, clinicians sometimes provide incomplete information about vaccines and, when met with reluctance, sometimes fail to follow-up or engage their patients in further discussion ( ((6))). Providers also report simply deferring discussions about vaccines when resistance is perceived ( ((76,80))). In this way, patient-provider communication may impact patients’ exposure to information about vaccines and their level of understanding.

Considering the reliance on providers as a trustworthy source of vaccine information ( ((81))), poor patient-provider communication may influence vaccine uptake in Black populations ( ((6,82))). The provision of information and appropriate recommendations about preventive care measures by healthcare providers is influential to the accessibility of services ( ((34,35))). The combination of insufficient information from healthcare providers, misinformation, and lower health literacy rates in Black populations all contribute to a lack of knowledge and the inability to make informed decisions about vaccinations ( ((6,29,75,76))). Moreover, patient-physician communication has been identified as an underlying factor in racial disparities in healthcare ( ((83))). Black patients consistently experience poorer communication, information provision, client participation, and participatory decision-making compared to White patients ( ((83,84))). Incidentally, patient-provider racial concordance is associated with improvements in these areas ( ((83))).

Issues of convenience, such as location, hours, cost, transportation, scheduling, wait times, and number of visits can serve as barriers or facilitators to the utilization of health services ( ((65,75–78,81,85))). Black people systematically have higher rates of unemployment, fewer opportunities, poorer compensation and benefits, and greater job instability than their White counterparts ( ((86))). Lower positions within their organizations and poorer benefits ( ((86,87))) result in lower utilization of paid time off and other forms of leave that would facilitate flexibility and convenience with respect to accessing care services ( ((88,89))). Additionally, comprehensive health insurance, which is typically associated with employment for many, directly impacts health service costs ( ((75,81,90))).

Trepidation toward public health interventions and the government is not unfounded, considering the persistent barriers to health services in Black communities and the tumultuous historical relationships between Black people and medicine ( ((88))). Significant incidents of abuse and mistreatment of Black populations by the medical community have been documented ( ((91))). The resulting mistrust now serves as a deterrent to health service utilization, especially preventive measures such as screenings and vaccinations ( ((88,90))). Additionally, medical mistrust, compounded with routine experiences of racism in accessing health services, cumulatively fosters hesitancy in healthcare utilization ( ((92,93))). It is reasonable that distrust and other beliefs regarding the healthcare system contribute to skepticism about vaccine development, concerns about safety, and perceptions of risk ( ((81,94))).

Black communities also have very strong cultural, religious, and family ties that inform their perspectives and worldviews on various issues related to health ( ((95))). In some instances, family, friends, and community leaders are a source of anti-vaccine sentiments; religious beliefs may object to vaccines or restrict vaccine utilization, and community conversations contribute to anti-vaccine propaganda ( ((81,96))). Dynamics within cultural, religious, and family structures also impact who has the authority to make decisions about vaccine uptake ( ((14,27,55))). Concerns about stigma or assumptions of health status from the community also further discourage engagement with vaccines ( ((55,65))).

Racial disparities in child and adult immunization rates have grown over time ( ((81,97,98))), in part due to this complex, multi-level influence on how Black populations engage with vaccinations. This review corroborates the existing literature on issues affecting rates of vaccine uptake and the prevalence of vaccine hesitancy in Black populations. Ultimately, identifying these influential factors within the Black population provides a stronger basis for developing policies, programs, and strategies to address vaccine hesitancy, increase vaccine uptake, and reduce racial health disparities.

### Implications for research, policy, and practice

Medical mistrust among African Americans is derived from historical, discriminatory, and harmful racial experiences associated with the healthcare system and government. This has led to avoidance of healthcare as a self-protective coping strategy. Black communities are more willing to be vaccinated when it is recommended by a trusted healthcare provider ( ((7,8,99))). Although evidence indicates that Black populations have higher rates of vaccine hesitancy, no research has systematically synthesized the factors driving this hesitancy. Future research should explore the association between vaccine hesitancy, cultural and behavioural patterns, the longer-term support that people who hesitate to get vaccines may need, and how best to provide that support. Vaccine hesitancy continues to have a significant impact on global health ( ((100))). For example, severe acute respiratory syndrome coronavirus 2 (SARS-CoV-2), the virus that causes COVID-19 first appeared in 2019 in Wuhan, China and then spread to nearly all countries of the world in only a few months ( ((101))). The high rate of infectivity of COVID-19 makes the estimated mortality rate and impact on the economy unprecedented. Importantly, the mortality rate from COVID-19 is 2.7 times higher in Black vs. White Americans ( ((102))). The reasons for the disproportionate effect on Black people likely stem from racism and other social determinants of health, such as low income, mass incarceration, infant mortality, limited healthcare access, and health-related conditions including heart disease, diabetes, stroke, kidney disease, respiratory illness, and HIV ( ((103))).

The devastating effects of COVID-19 on the Black community underscore the importance of governments establishing vaccination uptake policies and recommendations that would encourage greater vaccination coverage among the Black population. This measure will help reduce the Black community's death rate from disease and illness, keep coverage rates high, and curb the spread of disease. In the case of COVID-19 in particular, it will lessen the risk that the healthcare system will be overrun by the disease load from this virus and prevent the overstretch of limited healthcare resources. In addition, healthcare providers should make cultural safety a priority in their work to earn the Black community's trust and boost vaccination rates among this population. Efforts to implement interventions, such as home vaccination programs and initiatives to improve vaccine and health literacy, should particularly focus in individuals who are apprehensive about vaccination within Black communities. More Black people would be vaccinated if Black vaccinators were used ( ((104))).

### Strengths and weaknesses of this review

This scoping review employs rigorous methods, as it combines results from a large number of studies conducted using both qualitative and quantitative techniques. The research findings revealed many challenges to getting Black people vaccinated, which put them into the categories of structural, cognitive, behavioural, and sociocultural problems. This review provides an in-depth analysis of how trust in doctors, government bodies, and the vaccine creation process influences people’s decisions about vaccination. Furthermore, this review examines the issue on both personal and societal scales, providing a comprehensive understanding of the issue. The use of various types of vaccines in different regions enhances the effectiveness of vaccines in public health globally.

On the other hand, since most studies are cross-sectional, the review cannot correctly identify the reasons for increased vaccine hesitancy or its temporal changes. Despite a thorough assessment of how well the studies were carried out, the available evidence is taken at face value. Although thematic analysis involves considerable detail, the scoping review does not always combine the findings from studies to draw general conclusions or identify the most critical barriers.

## Appendix

Supplemental material is available upon request to the author: folajinm@ualberta.ca

Table S1: Characteristics of included studies
